# Treatment with Selective Serotonin Reuptake Inhibitors and Mirtapazine Results in Differential Brain Activation by Visual Erotic Stimuli in Patients with Major Depressive Disorder

**DOI:** 10.4306/pi.2009.6.2.85

**Published:** 2009-06-30

**Authors:** Won Kim, Bo-Ra Jin, Wan-Seok Yang, Kyuong-Uk Lee, Ra-Hyung Juh, Kook-Jin Ahn, Yong-An Chung, Jeong-Ho Chae

**Affiliations:** 1 Department of Psychiatry and Stress Research Institute, College of Medicine, Inje University, Seoul, Korea.; 2 Department of Psychiatry, The Catholic University of Korea, Seoul, Korea.; 3 Department of Medical Engineering, The Catholic University of Korea, Seoul, Korea.; 4 Department of Radiology, The Catholic University of Korea, Seoul, Korea.; 5 Department of Nuclear Medicine, The Catholic University of Korea, Seoul, Korea.

**Keywords:** Functional MRI, Selective Serotonin Reuptake Inhibitor, Mirtazapine, Sexual dysfunction

## Abstract

**Objective:**

The objective of this study was to identify patterns of brain activation elicited by erotic visual stimuli in patients treated with either Selective Serotonin Reuptake Inhibitors (SSRIs) or mirtazipine.

**Methods:**

Nine middle-aged men with major depressive disorder treated with an SSRI and ten middle-aged men with major depressive disorder treated with mirtazapine completed the trial. Ten subjects with no psychiatric illness were included as a control group. We conducted functional brain magnetic resonance imaging (fMRI) while a film alternatively played erotic and non-erotic contents for 14 minutes and 9 seconds.

**Results:**

The control group showed activation in the occipitotemporal area, anterior cingulate gyrus, insula, orbitofrontal cortex, and caudate nucleus. For subjects treated with SSRIs, the intensity of activity in these regions was much lower compared to the control group. Intensity of activation in the group treated with mirtazapine was less than the control group but grea-ter than those treated with SSRIs. Using subtraction analysis, the SSRI group showed significantly lower activation than the mirtazapine group in the anterior cingulate gyrus and the caudate nucleus.

**Conclusion:**

Our study suggests that the different rates of sexual side effects between the patients in the SSRI-treated group and the mirtazapine-treated group may be due to different effects on brain activation.

## Introduction

Our understanding of the brain substrates for sexual response is accumulating due to the development of functional imaging techniques such as positron emission tomography (PET) and functional magnetic resonance imaging (fMRI).^1^,^2^ Park et al.^3^ investigated relationships between brain activation and sexual response in 12 young males (mean age=23 years) with normal sexual function. They reported that the brain areas activated by erotic visual stimuli were the inferior frontal lobe, cingulate gyrus, insula, corpus callosum, caudate nucleus, globus pallidus, inferior temporal lobe, and thalamus. Arnow et al.^2^ developed an experimental paradigm to evaluate regional brain activation during sexual arousal that included an objective measure of penile tumescence and erotic visual stimuli, as well as presentation of neutral and visually stimulating control segments using fMRI technology. The major areas of activation associated with tumescence were the right insula/subinsular region, including the claustrum, caudate nucleus, putamen, cingulate gyrus, occipito-temporal area, and hypothalamus. A study comparing gender differences in sexual stimuli showed that only male subjects exhibited significant activation of hypothalamus.^4^

The impairment of sexual function in patients with depression is very common. One-third to one-half of patients with untreated depression have sexual difficulties manifested by decreased interest, and/or libido, erectile dysfunction or delayed ejaculation. Additionally, most antidepressants also create or increase sexual dysfunction. These side effects and sexual dysfunction increase with age.

Mirtazapine is a noradrenergic and a specific serotonergic antidepressant with a mode of action that is distinguished from popularly available antidepressants such as selective serotonin reuptake inhibitors (SSRIs). This drug is an antagonist of α2 receptors, and facilitates release of norepinephrine and serotonin.^5^ The enhancement of serotonergic neurotransmission is specifically mediated via 5-hydroxytryptamine (5HT)-1 receptors since mirtazapine is a postsynaptic serotonergic 5HT2 and 5HT3 antagonist.^6^ Data from clinical trials have shown that mirtazapine has an overall clinical efficacy similar to that of tricyclic antidepressants and has a relative absence of cholinergic, adrenergic, and serotonergic side effects.^7^-^9^ A series of studies suggest that the patients who have troublesome sexual side effects with SSRIs can show continued remission of depression as well as a return to satisfactory sexual functioning when switching to or augmenting with mirtazapine.^10^-^12^

Sexual dysfunction is one of the most common complaints amongst depressed patients. Not only depression itself but also other psychiatric illnesses or general medical conditions can cause sexual difficulties manifested by decreased interest, and/or libido, erectile dysfunction or delayed ejaculation. Additionally, most antidepressants induce or increase sexual dysfunction. While these side effects and sexual dysfunction increase with age,^13^ the SSRIs, venlafaxine, the tricyclic antidepressants, and monoamine oxidase inhibitors (MAOIs) are all associated with a decrease in sexual desire, impotence, delayed ejaculation, and anorgasmia. These drugs also can affect all phases of sexual activity: desire, arousal, orgasm, and ejaculation. However, several studies have demonstrated that the most widely-used antidepressants, SSRIs, are especially associated with higher rates of sexual dysfunction.^14^

Compared with SSRI, mirtazapine is reported to be less associated with sexual dysfunction. Although 5HT2 and 5HT3 antagonism are thought to underlie the decrease in sexual dysfunction, the precise mechanism is not yet clear. Nowadays, neuroimaging studies about the neural correlates with sexual function are gaining attention^15^,^16^ and pharmacoMRI may reveal differential brain activation between these two classes of antidepressants.

We carried out this study in order to observe whether there are differences in brain activation elicited by visual erotic stimuli in elderly patients with depression treated with SSRIs or mirtazapine, and to identify the different regions that are associated with sexual dysfunction.

## Methods

### Subjects and assessments

Twenty-three depressed patients currently on SSRI or mirtazapine treatment and twelve healthy controls participated in the study. The participants were all age-matched and had no prior psychiatric illness. All participants were heterosexual, right-handed middle-aged males from 40 to 60 years of age. In the control group, we excluded those who had a history of sexual arousal disorder or erectile dysfunction, and those with medical illness or history such as diabetes mellitus, hypertension, or other serious illness.

The subjects in the depression group were diagnosed with major depressive disorder by two board-certified psychiatrists according to the Diagnostic and Statistical Manual of Mental Disorders (DSM)-IV criteria. The Structured Clinical Interview, DSM-IV Axis I Disorders-Clinician Version,^17^ was assigned to all patients and controls. Those with medical illness or history such as diabetes mellitus, hypertension, or other serious illness were excluded.

The symptom score in the 17-Item Hamilton Rating Scale for Depression (HAM-D)^18^ and in the Clinical Global Impression-Severity of Illness (CGI-S)^19^ scale score was obtained for each subject in the depression group. Adverse side-effects of drug treatment were obtained at the time of enrollment. The absence of physical disorders and of any pharmacological treatment other than the antidepressants was checked through a medical examination.

All potential subjects were screened via a one-hour interview and were encouraged to fill out various questionnaires including the Brief Male Sexual Function Inventory (BSFI)^20^. The BFSI is a validated self-report measure on sexual functioning. The Inventory covers sexual drive and satisfaction, erectile function, ejaculatory function, as well as the problem assessment of drive, erection, and ejaculation. The item scaling ranges from 0 (no function, big problem, etc.) to 4 (good function, no problem, etc.) while the domain score is calculated by adding the item scores. Lower scores mean poorer function.

Eight patients in the SSRI group were taking paroxetine while four were taking fluoxetine. There were eleven patients in the mirtazapine group. The mean duration of depressive illness was 32±17.8 weeks in the SSRI group, and 36±14.9 weeks in the mirtazapine group. The mean duration of medication for the SSRI group was 11±4.9 weeks, and 10±5.4 weeks in the mirtazapine group. The duration of illness and the duration of medication between the two antidepressant groups were not different.

The selected subjects were thoroughly briefed on the design of the study, allowed to read and sign a written consent form before participating. The study protocol was approved by the institutional review board at St. Mary's Hospital, The Catholic University of Korea.

### Activation stimuli and MRI image acquisition

We presented a film clip that lasted for 14 minutes and 9 seconds to each subject. Each clip consisted of alternating segments of relaxing scenes (R), sports highlights (S), and sexually arousing erotic scenes (E) in the following order: S, R, E, R, E, R, S, R, S, R and E. The respective times for each segment in seconds were: 129, 60, 120, 30, 120, 30, 120, 30, 60, 30, 120 (seconds). The contents from the relaxing scenes were natural scenes such as the mountains and the valleys while the scenes from the sports highlights were various scenes from two-person competitions in the Olympic Games. There was one factor that had to be taken into consideration: Given data suggesting that subjects disengaged from emotionally stimulating visual material under fMRI conditions for a period of approximately 15 seconds, the S and E segments were not contiguous and were separated by a minimum of 30 s of R.^21^ The content of the erotic segments involved four types of sexual activities: rear-entry intercourse, intercourse with the female in the superior position, fellatio and sexual intercourse with the male in the superior position. Of the eight different segments depicting sexual activity, these four activities were associated with the highest level of perceived sexual arousal and penile erection in a sample of 40 healthy males. Additionally, in order to control for a possible anticipation effect, subjects were not aware of the ordering of the segments.

During the fMRI sessions, the film clips were presented to the subjects through a mirror located at the top of the head coil that receives video-images from outside of the magnetic room. Echoplanar images (EPI) were acquired on a 1.5 Tesla MRI system (Magnetom Vision Plus, Siemens, Erlangen, Germany). Thirty slices (5 mm thick) were acquired every 3.106 seconds in an inclined axial plane, aligned with the AC-PC axis. These T2-weighted functional images were acquired using an EPI pulse sequence (TR=0.6 msec, TE=60 msec, Flip 90, FOV=240 mm, Matrix=64×64). After functional scanning, high-resolution data were acquired via a T1-weighted 3D volume acquisition obtained using a gradient echo pulse sequence (TR=9.7 msec, TE=4 msec, Flip=12, FOV=240 mm, Matrix=200×256).

Data were analyzed using Statistical Parametric Mapping (SPM99, Wellcome Department of Cognitive Neurology, London, UK). Scans were realigned and spatially normalized using the standard Montreal Neurological Institute (MNI) template. Images were then convolved in space with a 3D isotopic Gaussian kernel (full width at half maximum, FWHM, of 8 mm) to improve the signalto-noise ratio and to accommodate residual variations in functional neuroanatomy that usually persist between subjects after spatial normalization. Effects at each and every voxel were estimated using the general linear model. Voxel values for each contrast yielded a statistical parametric map of the t statistic (SPMt) and subsequently transformed to the unit normal distribution, SPM {Z}. A "random-effects model" was implemented to produce the E (erotica) minus N (neutral) contrasts. This model was implemented within SPM99 using a multi-stage approach.

The hypothalamus, thalamus, anterior cingulate gyrus, occipitotemporal cortex, anterior temporal cortex, parietal cortex, amygdala, hippocampal formation, orbitofrontal cortex, ventral striatum, the claustrum, the nucleus accumbens, and the parietal lobules have been reported to show increased activation in response to sexually explicit films in male subjects.^2^-^4^,^22^ For each of the brain areas mentioned above, a set of coordinates was calculated by taking the average for each orthogonal axis X, Y and Z of reported Talairach coordinates.^23^ Predetermined regions of interest (ROI) were limited by spheres having a radius of 9 mm and for center, the calculated average reported coordinates. For these a priori ROIs, the height threshold was set at p<0.001 (z=3.09), uncorrected for multiple comparisons. For other brain areas, the height threshold was set at p<0.05, corrected for multiple comparisons.

## Results

Of the twelve subjects initially enrolled in the control group, ten subjects finished the fMRI study with reliable imaging data. One subject withdrew his consent and dropped out of the study as he experienced discomfort while in the MRI gantry during the scan. The other subject's head movement was out of the permitted range and the data were unreliable. The results from the remaining normal middle-aged subjects have been previously described.^24^

Of the subjects initially enrolled in the experimental groups, three subjects violated the study protocol (taking other antidepressants and sildenafil) between the periods of enrollment and fMRI scan. Because one subject's scan had marked noise due to technical reasons, the data were excluded from further analysis. We thus analyzed data for nine subjects undergoing treatment with SSRIs (6 paroxetine, 3 fluoxetine) and ten patients undergoing treatment with mirtazapine. There were no statistically significant differences between the two groups in baseline demographics and clinical data (Table 1). The daily mean dosages throughout the present study were 33.9 mg/day for the SSRI group and 39.8±10.0 mg/day for the mirtazapine group. None of the patients reported serious adverse events and were able to generally tolerate both treatments well. There were significant differences in the scores on the Brief Male Sexual Function Inventory (BSFI)^20^ between the control subjects and the depressive patient group treated with antidepressants. There was no significant differences in the BSFI scores between the SSRI treated group and the mirtazapine treated group.

**FIGURE 1:**
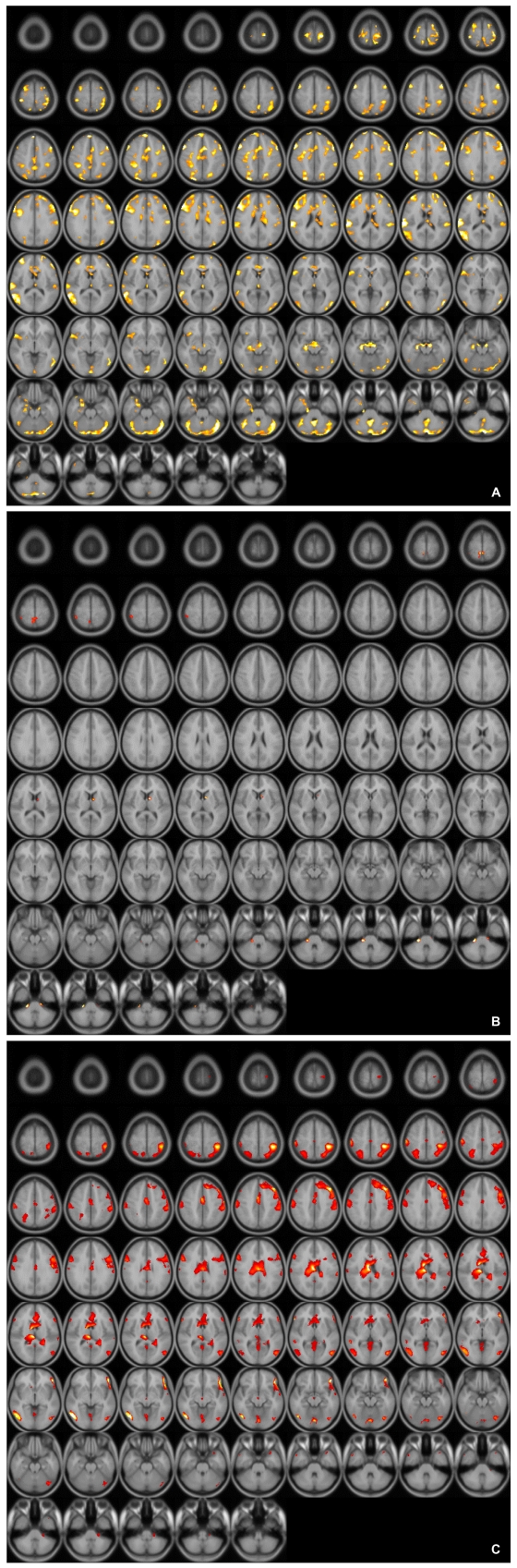
A: Axial view of activated brain areas by erotic visual stimuli in healthy controls. B: Axial view of activated brain areas by erotic visual stimuli in patients with depression who treated with SSRI. SSRI: selective serotonin reuptake inhibitor. C: Axial view of activated brain areas by erotic visual stimuli in patients with depression who treated with mirtazapine.

When the blood oxygen level dependent (BOLD) activity while viewing emotionally neutral film segments (S) in the control group was subtracted from that obtained while viewing erotic segments (E), there was a significant (p<0001, uncorrected) pattern of activation that represented brain regions that responded to the visual sexual stimuli in the control group, the SSRI group, and the mirtazapine group (Figure 1A, B and C). As shown in previous reports,^24^ healthy middle-aged male controls showed activation in the occipitotemporal area, anterior cingulate gyrus, insula, orbitofrontal cortex and caudate nucleus after viewing visual sexual stimuli (Figure 1A). Meanwhile, depressed patients treated with SSRI showed lower BOLD activity patterns than in the control group (Figure 1B). The brain activation elicited by sexual images in the depressed patients treated with mirtazapine also showed lower activation compared to that of the control group although less decreased than in the SSRI group (Figure 1C).

**FIGURE 2:**
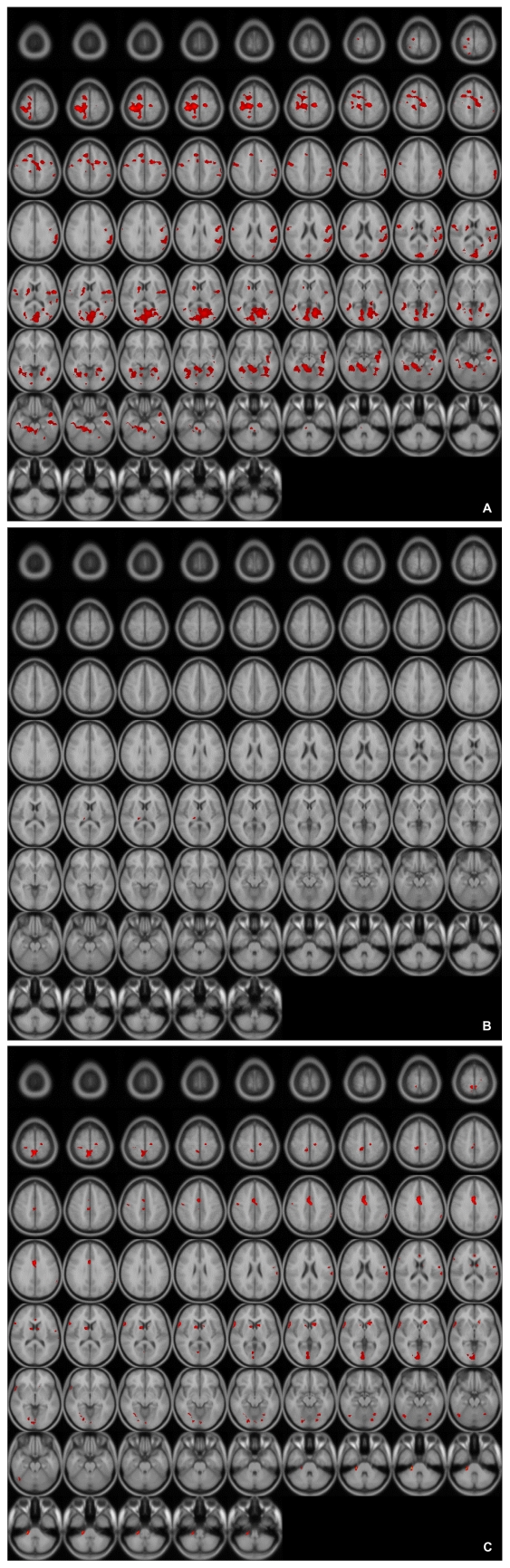
A: Differences of activated brain regions in response to sexually explicit stimuli between control and patients with SSRI treated depression (substraction images: normal healthy control minus patients with depression who treated with SSRI). SSRI: selective serotonin reuptake inhibitor. B: Differences of activated brain regions in response to sexually explicit stimuli between normal healthy control and patients with mirtazapine treated depression (substraction images: normal healthy control minus patients with depression who treated with mirtazapine). C: Differences of activated brain regions in response to sexually explicit stimuli between patients with mirtazapine treated depression and SSRI treated depression (substraction images: patients with depression who treated with mirtazapine minus patients with depression who treated with SSRI). SSRI: selective serotonin reuptake inhibitor

These patterns were more definitive when the BOLD activity elicited by the erotic visual stimuli in the SSRI group and the mirtazapine group was subtracted from that obtained from the control group. There were significant (p<0.05, corrected for multiple comparisons) differences in several brain regions activated in response to sexual visual stimuli between the control group and the patients treated with antidepressants. Superior frontal gyrus, postcentral gyrus and pons seemed to be less activated than in the controls amongst the patients in the SSRI group (Table 2, Figure 2A). For the patients in the mirtazapine group, right thalamus is activated significantly less than those in the control group (Table 3, Figure 2B). Additionally, when the BOLD activity for the SSRI group was subtracted from that of the mirtazapine group, the erotic image-elicited BOLD activity from the SSRI group showed significantly lower activation in the anterior cingulate gyrus and the caudate nucleus than that of the mirtazapine group (Table 4, Figure 2C).

## Discussion

Sexual dysfunction is one of the most frequent and disantidepressants such as SSRIs and serotonin norepinephrine reuptake inhibitors (SNRIs).^25^ These effects are associated with increased serotonergic tone and various serotonin receptors such as 5HT1a and 5HT2 receptors.^6^ A recent attempt to specify the detailed sexual inhibitory effects of serotonin showed that there is an inhibitory role of serotonin in the lateral hypothalamic area.^26^ This is consistent with the well-established inhibitory effects of SSRIs on sexual response.^27^ However the precise physiologic mechanisms that mediate this phenomenon are as yet unknown.

Our results showed that cortical areas such as right postcentral gyrus, left superior frontal gyrus and pons activated significantly less in depressed patients treated with SSRIs compared to the normal middle-aged healthy male subjects. As reported previously, the middle-aged healthy subjects showed less hypothalamic and thalamic activation compared to the younger men.^24^ Yang^28^ also examined brain activation from visual sexual stimuli amongst the depressed patients with sexual dysfunction and reported that hypothalamus as well as thalamus were both less activated than the normal control subjects. Therefore, we can suggest that the middle-aged depressive patients undergoing SSRI treatment suffer more sexual dysfunction due to less hypothalamic and thalamic activation as well as a decrease in cortical activation. However, since hypometabolism of the left prefrontal area was also reported amongst depressive patients,^29^ it is difficult to clarify whether hypoactivation of left superior frontal gyrus is due to the SSRI or the depression itself.

In our results, the BOLD activities in the patients with mirtazapine showed greater intensity than the SSRI group, among cingulate gyrus, right frontotemporal lobe, and left caudate head. These results suggest that patients taking SSRIs may have more difficulties in normal sexual functioning compared to patients taking mirtazapine since the cingulate gyrus and caudate nucleous are known to be important neuroanatomical regions for normal sexual function. Human sexual arousal is proposed to be a multidimensional experience comprised of four closely interrelated and coordinate components: cognitive, emotional, motivational, and physiological, that are all mediated by the brain.^22^,^30^ While caudate nucleus is reported to be correlated with the urge to perform hand-washing rituals in patients with obsessive-compulsive disorder, McGuire et al.^31^ noted that during viewing of an erotic film, subjects were simultaneously confronted with the urge to act and with the impossibility to do so, hence suggesting a role for the ventral striatum in the control of the motor expression of sexual arousal, that is, in withholding the motor output of sexual arousal. Such a role could be implemented through the anatomic projections that the striatum receives from the cognitive subdivision of the anterior cingulate cortex.^32^ Reiman et al.^33^ also provided some evidence that the anterior cingulate gyrus is involved in the conscious experience of emotion. In a functional neuroimaging study, the activation of the anterior cingulate gyrus was highly correlated with the levels of perceived sexual arousal associated with the perceived urge to perform sexual actions.^22^ The anterior cingulate is subdivided into "affect" and "cognition" components, assessing the motivational content of the internal and external stimuli and regulating context-dependent behaviors often engaged in responses associated with affect.^32^ According to this neurobehavioral view, the activation of the anterior cingulate cortex may reflect the maintenance of the correspondence between the sexual response and the affective value of the stimulus. Ferretti et al.^16^ also suggested that anterior cingulate had a major role in the every phase of male sexual arousal, while other brain region such as insula and hypothalamus are more activated in some phases. Activity in the anterior cingulate was also associated with sexual arousal of women in some studies.^34^,^35^ These results have been replicated in several studies using fMRI amongst healthy young subjects.^2^-^4^,^16^ Therefore, it suggests that the differential activation of cingulate gyrus and caudate nucleus obtained in this study reflects the different sexual dysfunctional effects of mirtazapine and SSRI.

We have found that fMRI during the presentation of sexually arousing stimuli in patients undergoing antidepressant therapy is useful for gaining a better understanding of the exact nature of the brain's response during the time of sexual arousal. It would also provide a potential means to objectively measure brain activation in response to sexually arousing stimuli.

Finally, the present study supported the notion that the different rates of incidence in sexual dysfunction amongst the patients treated with SSRIs and mirtazapine could reflect their different mechanisms of action in the brain. By and large, SSRI treatment results in more profound dysfunctional effects on the sexual activities within brain than does mirtazapine treatment. Thus our results support that mirtazapine is superior to SSRIs in the aspect of sexual side effect profiles.

An important issue is the general effect of antidepressant on blood flow. In fMRI, activations measured are an indirect reflection of neural activity but a direct reflection of the difference between the magnetic properties of oxygenated and deoxygenated hemoglobin or BOLD response. Theoretically, it is possible that serotonin influenced blood flow or BOLD changes in the present study. SSRIs and mirtazapine may decrease the activation of BOLD through serotonergic and histaminergic affinities, but this is a controversial point. SSRIs have been regarded as good tools to use in pharmacoMRI studies to manipulate the serotonergic system.^36^ SSRIs and mirtazapine in general have few cerebrovascular effects.^37^,^38^ Therefore, it is unlikely that general effects on the BOLD response caused by SSRIs and mirtazapine influenced the results.

There are several limitations of this study. First, a relatively small number of heterogeneous subjects participated in the study. We included patients taking either fluoxetine or paroxetine as the SSRI group although the two antidepressants are somewhat different pharmalogically. Second, we did not apply objective measures of sexual arousal such as penile tumescence. We assessed sexual function using only the BSFI questionnaire. In addition, we did not perform the BSFI questionnaire before starting the antidepressant treatment. Third, we did not include auditory stimuli in this paradigm. Although sound is important in sexual activation, we showed only visual stimuli for the simple paradigm. Fourth, the activated regions in the comparison between patients and controls could be due to depressive symptoms as well as the effect of medication at the same time. Therefore we cannot make clear that the different activation is due to medication. The different thresholds for a priori and other brain areas included in the analysis could skew the results. Because depression also causes sexual dysfunction, the lack of assessment excluding the effect of antidepressant is one of its limitations. However, we recruited the subjects who stated that their sexual dysfunction develops after taking the antidepressant. Additionally, we did not measure brain activity occurring before starting the treatment with the antidepressant. Nevertheless, we did find the difference in BOLD activities between the mirtazapine group and the SSRI group. The result may reflect the difference in sexual dysfunction profile between the two antidepressants. The regions that showed the differences in BOLD activities were similar to that of the brain regions known to be related with normal sexual function. Finally, although we did not find a direct correlation between the reported sexual dysfunction and the brain activations by fMRI study, our study was meaningful because it was the first attempt to directly compare the effect of SSRIs and mirtazapine on patterns of brain activation. As neuroscience has progressed, the relation between the patterns of brain activation and clinically relevant features has gathered much interest. Research such as this preliminary study will assist the exploration of the labyrinth of various psychiatric clinical problems.

We found differences in patterns of brain activation between SSRI treated- and the mirtazapine-treated groups after depressive patients viewed erotic visual stimuli. Our results showed that BOLD activity was less intense in the SSRI treated group than in the control group. The cingulate gyrus, right frontotemporal lobe, and left caudate head activated significantly more in response to erotic visual stimuli in the mirtazpine group than the SSRI group. Therefore, our study suggests that different rates of sexual adverse events amongst the patients in the SSRI-treated group and the mirtazapine-treated group may be due to differential effects of these drugs in the brain. Furthermore, the results indicate the neural substrates underlying the relatively less profound sexual dysfunction experienced by patients on mirtazapine as opposed to SSRIs.

## Figures and Tables

**TABLE 1:**
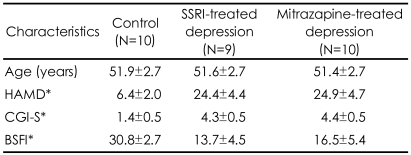
Demographic and clinical characteristics of normal control subjects, depressive patients treated with selective serotonin reuptake inhibitors (SSRI) and mirtazapine (mean±SD)

^*^p<0.001, Kruskal-Wallis Test [Posthoc test by Man-Whitney U test; HAMD-Control <SSRI group, mirtazapine group (p<0.001); CGI-S-Control <SSRI group, mirtazapine group (p<0.001), BSFI-Control >SSRI group, mirtazapine group (p<0.001)]. HAM-D: Hamilton Rating Scale for Anxiety, CGI-S: Clinical Global Impression-Severity of Illness, BSFI: Brief Male Sexual Function Inventory

**TABLE 2:**
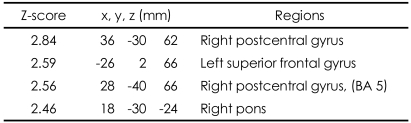
Differences of activated brain regions in response to sexually explicit and emotionally neutral visual stimuli between normal healthy control subjects and patients with depression who treated with SSRI (substraction: normal healthy control minus patients with depression who treated with SSRI)

SSRI: selective serotonin reuptake inhibitor, BA: Brodmann's Area

**TABLE 3:**
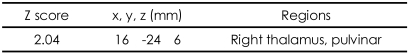
Differences of activated brain regions in response to sexually explicit and emotionally neutral visual stimuli between normal healthy control subjects and patients with depression who treated with mirtazapine (substraction: normal healthy control minus patients with depression who treated with mirtazapine)

**TABLE 4:**
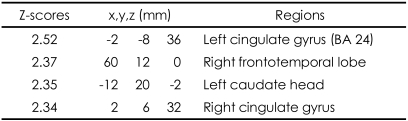
Differences of activated brain regions in response to sexually explicit and emotionally neutral visual stimuli between patients with depression who treated with mirtazpine and who treated with SSRI (substraction: patients with depression who treated with mirtazapine minus patients with depression who treated with SSRI)

SSRI: selective serotonin reuptake inhibitor
